# Collection of *in vivo* Capacitated Sperm from Different Locations Along the Reproductive Tract of Time-Mated Female Mice by Microdissection

**DOI:** 10.21769/BioProtoc.4193

**Published:** 2021-10-20

**Authors:** Lukas Ded, Jean-Ju Chung

**Affiliations:** 1 Department of Cellular & Molecular Physiology, Yale School of Medicine, New Haven, USA; 2 Laboratory of Reproductive Biology, Institute of Biotechnology, Czech Academy of Sciences, BIOCEV, Vestec, Czech Republic; 3 Department of Obstetrics, Gynecology, and Reproductive Sciences, Yale School of Medicine, New Haven, USA

**Keywords:** Mice, Oviduct, Sperm, Microdissection, Timed mating, Microscopy

## Abstract

Mammalian sperm cells are not capable of fertilizing an egg immediately after ejaculation; instead, they must gradually acquire the capacity to fertilize while they travel inside the female reproductive tract. Sperm cells are transported by the muscular activity of the myometrium to the utero-tubal junction (UTJ) before entering the oviduct where they undergo this physiological process, termed capacitation. Since the successful emulation of mammalian sperm capacitation *in vitro*, which led to the development of *in vitro* fertilization techniques, sperm capacitation and gamete interaction studies have been mostly carried out under *in vitro* conditions. Sperm cells are typically incubated *in vitro* for up to several hours at a concentration of more than 1 million cells per milliliter in the capacitation media inside a 37°C incubator with 5% CO_2_, mimicking the tubal fluid composed of serum albumin, bicarbonate, and Ca^2+^. The resultant sperm are functionally and molecularly heterogeneous with respect to acrosome reaction, motility, and phosphorylation. By contrast, *in vivo* sperm capacitation occurs in a time- and space-dependent manner, with limits on the number of capacitating sperm in the oviduct. The small number of sperm at the fertilization site *in vivo* are highly homogeneous and uniformly capable of fertilization. This discrepancy makes the degree of correlation between the changes observed from *in vitro* capacitation as a population average and the fertilizing capacity of sperm less clear. To overcome this issue, we used CLARITY tissue clearing to visualize sperm directly inside the female tract *in situ* and isolated sperm capacitated *in vivo* from the oviducts of the female mice after timed mating (Ded *et al.*, 2020). Here, we present a step-by-step protocol to collect *in vivo* capacitated sperm by detailing a microdissection technique and subsequent preparation steps for fluorescent imaging. The advantage of the microdissection technique over *in vitro* capacitation is the ability to collect physiologically segregated, homogeneous sperm populations at different stages of capacitation. Compared to CLARITY, this technique is more straightforward and compatible with a broader spectrum of antibodies for downstream imaging studies, as it allows the researcher to avoid a potentially high background from non-sperm cells in the tissue. The disadvantage of this technique is the potential contamination of the isolated sperm from different regions of the oviduct and disruption of the fine molecular structures (*e.g*., CatSper nanodomains) during sperm isolation, especially when the preparation is not performed swiftly. Hence, we suggest that the combination of both *in situ* and *ex vivo* isolated sperm imaging is the best way how to address the molecular features of *in vivo* capacitated sperm.

## Background

The discovery of sperm capacitation occurred in the 1950s when researchers failed to fertilize eggs *in vitro* by directly adding sperm to the oocytes without any prior incubation in media of similar composition close to the oviductal fluid (Austin, 1951; Chang, 1951). Since then, a vast majority of the existing information about the physiological and molecular processes surrounding capacitation was obtained using *in vitro* systems, which were first used in the 1970s for the conception of the first child through *in vitro* fertilization (IVF) (Steptoe and Edwards, 1976). Nowadays, up to 10% of children in developed countries are conceived by assisted reproductive technologies such as IVF and intracytoplasmic sperm injection (ICSI). With this development, there is an even greater need to obtain detailed knowledge about the natural capacitation process and to understand its differences from *in vitro* capacitation. This protocol enables researchers to study the molecular features of sperm at the different stages of the capacitation process by isolating sperm from different locations along the reproductive tract (UTJ, isthmus, and ampulla) of time-mated female mice. This protocol can also be applied to recover freshly ejaculated sperm from mice (Li *et al.*, 2015; Chung *et al.*, 2017) as epididymal sperm are normally used for mouse experiments, as opposed to ejaculate sperm for large domestic animals or humans.

## Materials and Reagents

1. Mini Petri dishes, 30 mm (Sigma-Aldrich, catalog number: SLW1480/12D)
2. Microsurgical scalpel (Stab Knife, 22.5 Degree, Straight; Sharpoint, catalog number: 72-2201)
3. Glass microcapillaries (50-μl calibrated pipets; Drummond, catalog number: 2-000-050)
4. Aspirator tube assemblies for calibrated microcapillary mouth pipette (Sigma-Aldrich, catalog number: A5177-5EA)
5. Syringe filter (0.2 μm; Pall Life Sciences, catalog number: PN 4612)
6. Epredia^TM^ PTFE Diagnostic Slides, 3-well (Thermo Fisher Scientific, catalog number: 10632391)
7. Epredia^TM^ PTFE Diagnostic Slides, 8-well (Thermo Fisher Scientific, catalog number: 10727951)
8. Animals: C57BL/6J female mice (3-5 weeks old)
9. Gonadotropin from pregnant mare serum; PMSG (Sigma-Aldrich, catalog number: G4877)
10. Chorionic gonadotropin human; hCG (Sigma-Aldrich, catalog number: CG10)
11. Fibronectin [Sigma-Aldrich, catalog number: F1141-2MG (solution; from bovine plasma) or 11051407001 (powder; from human plasma)]
12. M2 medium (Sigma-Aldrich, catalog number: M7167)
13. Mineral oil (Sigma-Aldrich, catalog number: M8410)
14. PBS (Sigma-Aldrich, catalog number: P4417)

## Equipment

1. An animal room with a 12-hour light/dark cycle, red-light option, and a light timer that allows the researcher to shift the light schedule to be more compatible with normal working hours
2. Stereo microscope (Nikon, model: SMZ1270)

## Procedure

- Timed mating of female mice in the estrous stage
  1. For timed mating, hyper-stimulated (super-ovulated) or non-stimulated female mice can be used. The hyper-stimulation procedure increases the chance of successful timed mating.
    - Hyper-stimulation: Hyper stimulate female mouse (22-24 days old optimally but up to 35 days old) with an intraperitoneal (i.p.) 5-IU injection of PMSG, followed by a 5-IU i.p. injection of hCG 48 h after PMSG. Calculate the time of both injections to start timed mating 12-14 h after the hCG injection. The protocol can be modified according to the animal stock provider’s suggestions (*e.g*., The Jackson Laboratory; https://www.jax.org/).
      Alternatively, the estrous cycle can be determined by examining washes of cell smears of the vagina to increase the success rate of the timed mating.
    - Determining the stage of the estrous cycle in female mice is described in detail inChamplin *et al.* (1973) and Caligioni (2009).
  2. Introduce a male to a female mouse housed in the single cage during the last dark hour of the 12 h long dark cycle (30 min to 1 h). Work under red light when handling mice for mating to not disrupt the light/dark cycle.
  3. Check the presence of vaginal plugs as soon as the light cycle begins (set this time as 1 h post-coitus). Females with plugs can be used for the experiment at a desired time point (*i.e*., 3 h or 7 h post-coitus). Save females without plugs as they can be used for another run of timed mating.
  4. Hormone injection and mating time should be customized according to the availability of the animal room with flexible light/dark cycle adjustment and the experimenter’s schedule. The following example is based on the lack of such facility and flexible schedule of an experimenter: In a facility with a 12-h light/dark cycle with the light on at 7 am and off at 7 pm, PMSG can be injected on Day 1 at 6 pm, followed by 6 pm hCG injection on Day 3. At 6 am (12 h after hCG) on Day 4, start timed mating under red light without disrupting the light/dark cycle by introducing a male to females. When the light is on at 7 am, the females are allowed to mate for 2 h. In the next 30 min or so, check the plug and sacrifice the plugged females at 9:30-10:30 am for collecting *in vivo* capacitated sperm from 3-4 h post-coitus or 4:30-5:30 pm for collection *in vivo* capacitated sperm at 7-8 h post-coitus.
- Microdissection and isolation of *in vivo* capacitated sperm
  1. After the sperm remain inside the female reproductive tract for a sufficient duration to allow *in vivo* capacitation, sacrifice the female mice with the confirmed vaginal plugs either by CO_2_ asphyxiation or cervical dislocation.
  2. Under the well-lit stereo microscope, grip the fat pad around the ovary and stretch gently to clearly identify the membranous boundary between the ovary and the oviduct (*line 1*) and the oviduct and the uterus (*line 2*) (**Figure 1**).
  3. Carefully cut through the membrane around the proximal oviduct to slightly de-coil the oviduct first (*line 2*), followed by the membrane around the bursa and just outside infundibulum (line 1), and lastly, cut through the uterus just outside UTJ (*line 3*) (**Figure 1**).
    **Figure 1.**
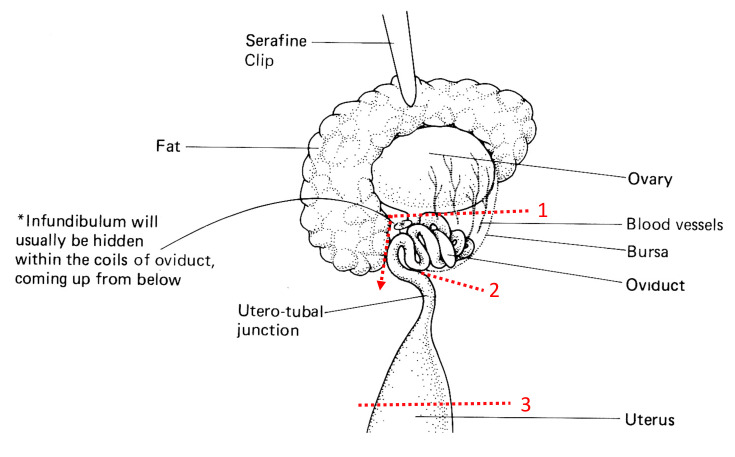
Scheme of mouse female reproductive tract dissection. Drawing slightly modified from Nagy (2003) in *Manipulating the Mouse Embryo*.
  4. The trimmed female tract (*right*) and the number and distribution of *in vivo* capacitated sperm (*left*) will look like **Figure 2A.
**
  5. (Optional) If Acr-EGFP or Su9-DsRed/Acr-EGF mice are used, the distribution and number of sperm can be validated under the fluorescent microscope (**Figure 2B**).
    **Figure 2.**
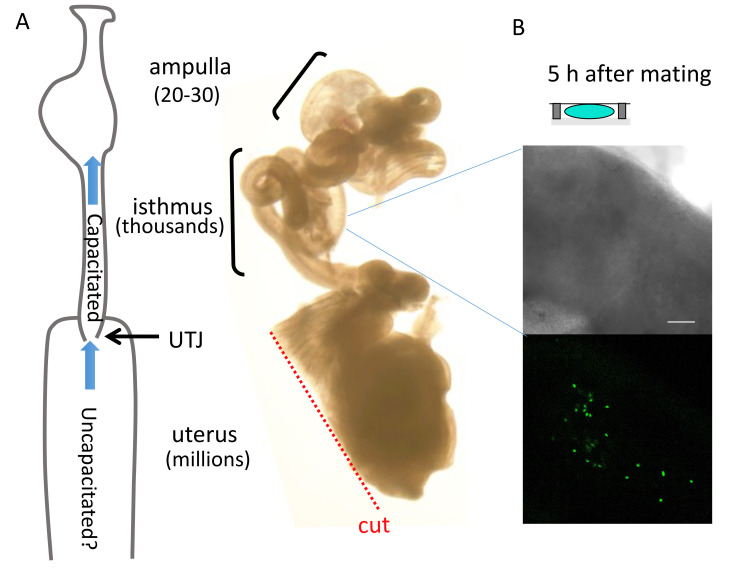
Distribution of sperm along the female reproductive tract. (A-B) Number of sperm recovered from each region of the female tract, aligned with an actual example image of a dissected female tract as described. Scale bar, 50 μm. (C) Validating location and number of Acr-EGF sperm in the isthmus with fluorescence microscopy.
  6. Place individual trimmed tract into a 0.3 ml-droplet of M2 media under mineral oil (**Figure 3A**) and further dissect them carefully with a microsurgical scalpel into ampullar, middle isthmus (2-3 mm), and UTJ parts (**Figure 3B**).
  7. Immediately place each part into an individual a 100-μl droplet of M2 media in a mini-Petri dish under mineral oil and gently press to release sperm (**Figure 3B**).
    **Figure 3.**
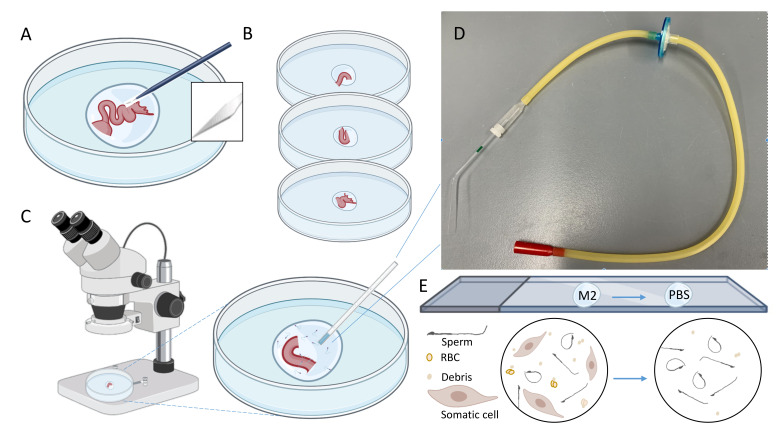
Microdissection and aspiration of sperm from different regions of the female reproductive tract. (A) Oviduct dissected by microsurgical scalpel placed into a 0.3-ml M2 droplet. (B) Placing individual oviductal parts into a 0.1-ml M2 droplet. (C) Observation under stereo microscope and picking sperm by glass microcapillary. (D) A setup of mouth aspiration pipette with a glass capillary tube and a filter. (E) A schematic cartoon representation of visible components of the droplet under the microscope before and after additional transfer/washing.
  8. Observe the droplets (optimally by three persons) under (stereo) microscopes (*e.g*., for 10 min) and pick individual sperm with an aspirator connected to a filter in the middle and 50-ul glass microcapillary by mouth pipetting (**Figure 3C and 3D**).
  9. Depending on the speed of dissection and handling of the tissue, there will be other cell types, such as discharged epithelial cells and red blood cells (RBC), and debris together with sperm cells (**Figure 3E**). As sperm cells are smaller than these somatic cells (except fine debris), transferring the liquid to the 2^nd^ droplet by avoiding these contaminants can help to obtain a preparation more enriched with sperm cells.
- Slide preparation
  1. Coat the surface of the slides with fibronectin. (A) Method A uses the fibronectin solution from bovine plasma: take 50-100 µl directly from the vial and spot onto the surface multiples times, making a domed solution with the desirable size. After 2-5 min when the edge of the dome is about to dry, rinse the coating solution with water. The slide/coverslip is ready to use for the next step. Method A does not require additional incubation time. (B) Method B prepares fresh fibronectin solution from fibronectin powder from human plasma. Reconstitute in sterile water by incubating for 30 min at 37°C to yield 1 mg/ml stock solution. Make small aliquots and keep frozen at -20°C until use. Prepare a fresh working solution (50 µg/ml in water) from an aliquot and apply 50 µl per well (3-well slide) or 10 µl per well (8-well slide). Incubate the slide/coverslip in a humid chamber for ~1 h. Rinse the coating solution with water and keep the slides in the chamber prior to sperm deposition. The time between the coating and sperm deposition should be as short as possible. Ideally, the whole procedure should be tested to optimize the cell attachment and reduce the background for the subsequent immunofluorescent microscopy due to the lot-to-lot variation of fibronectin.
  2. Place the picked-up sperm to 10 µl PBS droplet on the fibronectin-coated 8-well Teflon slides (**Figure 4A**: At a minimum, an average of 10 sperm per sample should be obtained). If too much tissue material is transferred by micropipette, a PBS washing step can be added (**Figure 3E**).
    **Figure 4.**
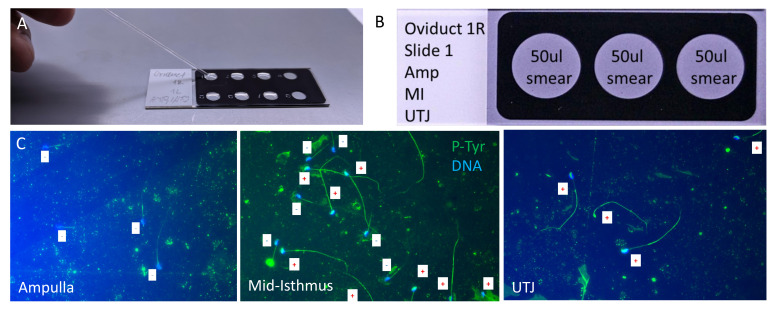
Slide preparation for downstream experiments and example images. (A) Sperm transfer by glass micropipette into 8-well Teflon microdroplet slides. (B) An example of setting up a 3-well Teflon smeared slide.
  3. Subject the rest of the material to finer dissection, remove macroscopic pieces manually, mix the rest of the material with PBS (material/PBS 1:10), and smear it on the fibronectin-coated 3-well Teflon slides (**Figure 4B**: ~30-50 µl per well).

## Data analysis

Obtained microscopic slides can be subjected to classical procedures for immunofluorescent staining, followed by confocal or super-resolution imaging. Shown here is an example of raw data (**Figure 4C**) used for P-Tyr quantification shown inDed *et al.* (2020) to illustrate a downstream experimental outcome.

## Notes

Mice hyperstimulation and vaginal smear analysis procedures are complex general protocols, and their detailed description can be found in provided references/links.
